# Towards patient-led follow-up after curative surgical resection of stage I, II and III colorectal cancer (DISTANCE-trial): a study protocol for a stepped-wedge cluster-randomised trial

**DOI:** 10.1186/s12885-023-11297-0

**Published:** 2023-09-07

**Authors:** Hidde Swartjes, Seyed M. Qaderi, Steven Teerenstra, Jose A. E. Custers, Marloes A. G. Elferink, Bob J. van Wely, Jacobus W. A. Burger, Wilhelmina M. U. van Grevenstein, Peter van Duijvendijk, Emiel G. G. Verdaasdonk, Marnix A. J. de Roos, Veerle M. H. Coupé, Geraldine R. Vink, Cornelis Verhoef, Johannes H. W. de Wilt

**Affiliations:** 1 Department of Surgery, Radboud university medical center, 6500 Nijmegen, The Netherlands; 2 Department for Health Evidence, Radboud university medical center, Nijmegen, The Netherlands; 3 Department of Medical Psychology, Radboud university medical center, Nijmegen, The Netherlands; 4 Department of Research and Development, Netherlands Comprehensive Cancer Organization, Utrecht, The Netherlands; 5 Department of Surgery, Ziekenhuis Bernhoven, Uden, The Netherlands; 6 Department of Surgery Catharina Hospital, Eindhoven, The Netherlands; 7 Department of Surgical Oncology, University Medical Center Utrecht, Utrecht University, Utrecht, The Netherlands; 8 Department of Surgery, Gelre Hospitals, Apeldoorn, The Netherlands; 9 Department of Surgery, Jeroen Bosch Hospital, ’s-Hertogenbosch, The Netherlands; 10 Department of Gastrointestinal Surgery and Surgical Oncology, Rijnstate Hospital, Arnhem, The Netherlands; 11 Department of Epidemiology and Data Science Amsterdam Public Health, Amsterdam UMC, Vrije Universiteit Amsterdam, Amsterdam, The Netherlands; 12 Department of Medical Oncology, University Medical Center Utrecht, Utrecht University, Utrecht, The Netherlands; 13 Department of Surgical Oncology and Gastrointestinal Surgery, Erasmus MC Cancer Institute, Rotterdam, The Netherlands

**Keywords:** Follow-up studies, Colorectal neoplasms, Surveillance, Telemedicine, Aftercare

## Abstract

**Background:**

Colorectal cancer (CRC) is among the most frequently diagnosed cancers. Approximately 20–30% of stage I-III CRC patients develop a recurrent tumour or metastases after curative surgical resection. Post-operative follow-up is indicated for the first five years after curative surgical resection. As intensified follow-up after curative surgical resection has shown no effect on survival, patient organisations and policy makers have advocated for a more patient-centred approach to follow-up. The objective of this study is to successfully implement patient-led, home-based follow-up (PHFU) in six hospitals in The Netherlands, with as ultimate aim to come to a recommendation for a patient-centred follow-up schedule for stage I-III CRC patients treated with surgical resection with curative intent.

**Methods:**

This study is designed as a stepped-wedge cluster-randomised trial (SW-CRT) in six participating centres. During the trial, three centres will implement PHFU after six months; the other three centres will implement PHFU after 12 months of inclusion in the control group. Eligible patients are those with pT2-4N0M0 or pT1-4N1-2M0 CRC, who are 18 years or older and have been free of disease for 12 months after curative surgical resection. The studied intervention is PHFU, starting 12 months after curative resection. The in-hospital, standard-of-care follow-up currently implemented in the participating centres functions as the comparator. The proportion of patients who had contact with the hospital regarding CRC follow-up between 12–24 months after curative surgical resection is the primary endpoint of this study. Quality of life, fear of cancer recurrence, patient satisfaction, cost-effectiveness and survival are the secondary endpoints.

**Discussion:**

The results of this study will provide evidence on whether nationwide implementation of PHFU for CRC in The Netherlands will be successful in reducing contact between patient and health care provider. Comparison of PROMs between in-hospital follow-up and PHFU will be provided. Moreover, the cost-effectiveness of PHFU will be assessed.

**Trial registration:**

Dutch Trail Register (NTR): NL9266 (Registered on January 1st, 2021).

**Supplementary Information:**

The online version contains supplementary material available at 10.1186/s12885-023-11297-0.

## Background

Colorectal cancer (CRC) is the third most common cancer worldwide [1]. Approximately 12,000 patients are annually diagnosed with CRC in the Netherlands [2]. Over the years, overall survival (OS) of CRC patients has improved significantly due to earlier diagnosis, and improved treatment of the primary tumour and metastatic disease [3, 4]. After curative-intent surgical resection of the primary tumour, approximately 20–30% of stage I-III CRC patients develop a recurrent tumour or metastases [5–8]. A part of this group of patients with recurrent CRC can be treated curatively. The general assumption is that because early detection of recurrence leads to increased rates of curative treatment, [9] the OS of patients in follow-up will increase as well. However, intensified follow-up after curative surgical resection has not shown an effect on OS [10].

At present, surgically resected stage I-III CRC patients receive in-hospital follow-up for five years, and five-year relative survival for Dutch stage I-III CRC patients is 65–95% [11]. The follow-up conform Dutch guidelines encompasses a standard protocol for surgically resected stage I, II and III CRC patients with carcinoembryonic antigen (CEA) measurements every three to six months during the first two years, and every six to 12 months during the third, fourth and fifth year. A computed tomography (CT) scan of the thorax and the abdomen is indicated at 12 months after curative surgical resection [12]. The present follow-up guideline was presented in December 2020. Prior to this revision, CT scans and/or ultrasounds had a more prominent role in the CRC follow-up, combined with regular CEA measurements. The frequency of colonoscopies did not change.

A systematic review by Jeffery et al. focused on the different follow-up strategies for non-metastatic CRC patients, and showed that intensive follow-up was associated with fewer interval recurrences (relative risk [RR]: 0.59, 95% CI: 0.41; 0.86) and that the rate of salvage surgery increased with intensive follow-up (RR: 1.98, 95% CI: 1.53; 2.56). They however concluded that there is no evidence for OS benefit for intensified follow-up after curative surgical resection (hazard ratio: 0.91, 95% confidence interval [CI]: 0.80; 1.04) [10]. Several large randomised controlled trials were included in this systematic review, such as the CEAwatch trial, [13] the FACS trial, [14] the COLOFOL trial, [15] and the GILDA trial [16]. Combined, abovementioned studies have provided the scientific basis for the present Dutch guideline on CEA-guided follow-up for curatively treated stage I-III CRC patients.

A narrative review by Qaderi et al. showed that hospital-based CRC follow-up is common and associated with high patient satisfaction, but that there is also a willingness of patients and health care providers for alternative CRC follow-up, including general practitioner-led or patient-led, home-based follow-up (PHFU) [17]. Qaderi et al. studied the implementation of such a PHFU plan at the Radboud university medical center, Nijmegen, The Netherlands [18]. One year after implementation, no statistically significant differences in health-related quality of life (HRQoL) or fear of cancer recurrence were found. Patient satisfaction with the PHFU plan was rated with a mean of 7.5 out of 10 (standard deviation: 1.3). Also, the PHFU showed a considerable cost-saving compared to the previous follow-up plan, i.e., a total cost of €14,880 versus €37,288 per year for the included patients (*N* = 118). Both studies by Qaderi et al. showed that there is a clear potential for PHFU. Accordingly, extension of scientific knowledge on this setting of CRC follow-up is desirable. This knowledge can be generated by means of an implementation trial of PHFU for CRC in a research setting.

This study aims to successfully implement PHFU for CRC in six hospitals in the Netherlands, and to evaluate the outcomes by making use of the infrastructure of the Prospective Dutch CRC cohort (PLCRC) [19, 20]. After diagnosis of CRC, patients can be informed about PLCRC and asked informed consent for the different components: use of medical data, and optionally questionnaires on patient reported outcome measures (PROMs), withdrawal of additional blood samples, participation in other (sub)studies and to be informed about coincidental findings.

The design of this study is a stepped-wedge cluster-randomised trial (SW-CRT). Patients will be followed between 12 and 24 months after curative surgical resection. Patient data collected in this period will be used to assess the study outcomes. The following outcomes will be compared between condition A and condition B: proportion of patients who had contact with the hospital regarding CRC follow-up during the study period, HRQoL, fear of cancer recurrence, anxiety, depression and distress, cost-effectiveness, and OS. Combined, these outcomes will determine the success of implementation. The final objective of this study is to come to a recommendation for a patient-centred follow-up schedule for stage I-III CRC patients treated with surgical resection with curative intent.

## Methods/design

### Study setting

**Fig. 1:**
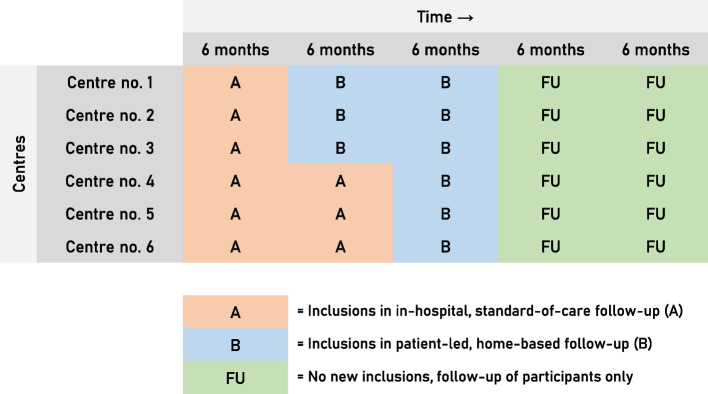
DISTANCE-trial study design

The study will be conducted in six hospitals in The Netherlands. The participating centres have no previous experience with PHFU for CRC. Five hospitals are non-academic teaching hospitals (Bernhoven Hospital, Catharina Hospital, Gelre Hospitals, Jeroen Bosch Hospital, Rijnstate Hospital). One hospital is a university medical centre (University Medical Center Utrecht). The six participating centres act as clusters. The study features two conditions: condition A (in-hospital, standard-of-care follow-up) and condition B (PHFU). All six participating centres will start including patients in condition A. Three centres will perform the switch from condition A to condition B after six months of participation; the other three centres will perform the switch from condition A to condition B after 12 months of participation. The switch will be performed without a transition period. A graphical overview of the study design and setting is depicted in Fig. 1.

### Eligibility criteria

All patients who underwent surgical resection with curative intent for pT2-4N0M0 (i.e., stage I-II) or pT1-4N1-2M0 (i.e., stage III) CRC in one of the six participating centres and who are disease-free at 12 months after curative resection are eligible for inclusion. Patients with pT1N0M0 CRC are not part of the study population, as oncological follow-up is not required for these patients, according to the current guideline [21] .

#### Inclusion criteria

In order to be eligible to participate in this study, a subject must meet all of the following criteria:

- Patient has pT2-4N0M0 or pT1-4N1-2M0 colorectal carcinoma treated with surgical resection with curative intent;
- Patient is aged ≥ 18 years;
- Patient gave informed consent for PLCRC;
- Patient is disease-free at 12 months after resection; and
- Patient will receive follow-up in the participating center beyond 12 months after resection.

#### Exclusion criteria

A potential subject who meets any of the following criteria will be excluded from participation in this study:

- Patient has macroscopically incomplete resections (R2);
- Patient needs in-hospital follow-up longer than 12 months postoperatively (e.g., patients with a severely complicated postoperative course, or patients enrolled in other studies that require in-hospital follow-up consultations); or
- Patient has confirmed hereditary CRC.

### Interventions

After diagnosis of CRC, patients can be informed and included in PLCRC. If a patient gives informed consent, their PLCRC key study number is matched with their key number in the Netherlands Cancer Registry (NCR). PROMs are collected with questionnaires. PLCRC sends out these questionnaires at predefined time points from the moment of inclusion in PLCRC. For this study, specific questionnaires are added to the package of questionnaires to assess a possible difference in PROMs between condition A and condition B.

Prior to inclusion, patients have already received 12 months of follow-up care after their curative surgical resection. At the 12-month time-point, patients will undergo a diagnostic CT thorax-abdomen, colonoscopy and CEA measurement according to the current guideline. These results will be discussed at an outpatient clinic visit and will serve as baseline data for this study. This outpatient clinic visit will also be the start of the study for patients. Patients can still be included in PLCRC at this visit. According to the condition of the participating centre at the time of inclusion (A or B), a patient’s follow-up trajectory will differ corresponding to that condition.

#### In-hospital, standard-of-care follow-up (condition A)

Between 12 and 24 months after curative surgical resection, patients in follow-up under condition A will receive follow-up according to the current standard-of-care follow-up schedule in the participating centre. It is possible that this follow-up schedule deviates slightly from the national guideline.

#### Patient-led, home-based follow-up (condition B)

Before or during their outpatient clinic visit at 12 months after curative surgical resection, patients who will receive follow-up under condition B will receive written information on the PHFU. The treating physician/nurse practitioner will educate the participant on the PHFU and consecutively start the PHFU. If patients are not eligible for PHFU, they will receive in-hospital follow-up.

Participants will have their blood samples for the CEA measurement drawn at their general practitioner, the local diagnostic laboratory or the hospital. Preferably, patients will have their blood samples drawn as close to home as possible.

In principle, participants will receive notification by (e-)mail or text-message that they will have to perform their blood sampling for the CEA measurement. Reminders will be sent when the patient has missed the CEA measurement.

**Fig. 2:**
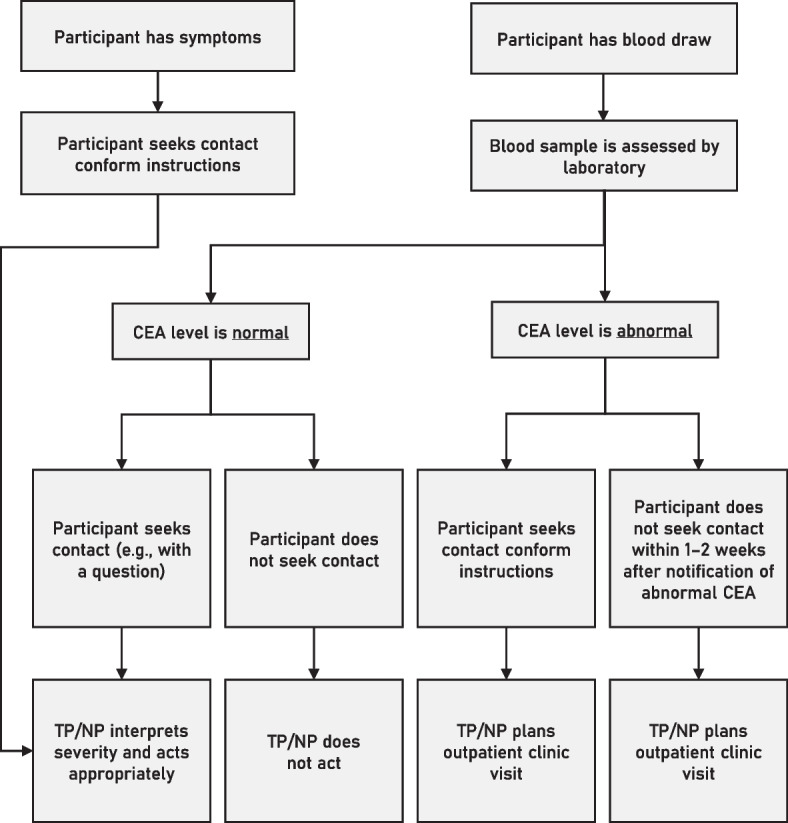
Flowchart of contact between participant and health care provider during patient-led, home-based follow-up. CEA; carcinoembryonic antigen. TP; treating physician. NP; nurse practitioner

The patient will evaluate their own CEA level through the online patient platform portraying their test results. If the CEA level is normal, the participant is instructed not to act. If the CEA level is abnormal or the participant experiences symptoms, the participant is instructed to seek contact with their treating physician/nurse practitioner. If the participant does not seek contact with their treating physician/nurse practitioner, but the treating physician/nurse practitioner notices an abnormal CEA level, the participant will be contacted. Due to this construction, abnormal CEA levels will always lead to contact between patient and the treating physician/nurse practitioner. This construction is visualised in Fig. 2. If a participant wants to plan an outpatient clinic visit, they can always contact their treating physician/nurse practitioner – even if their CEA levels are normal.

**Fig. 3:**
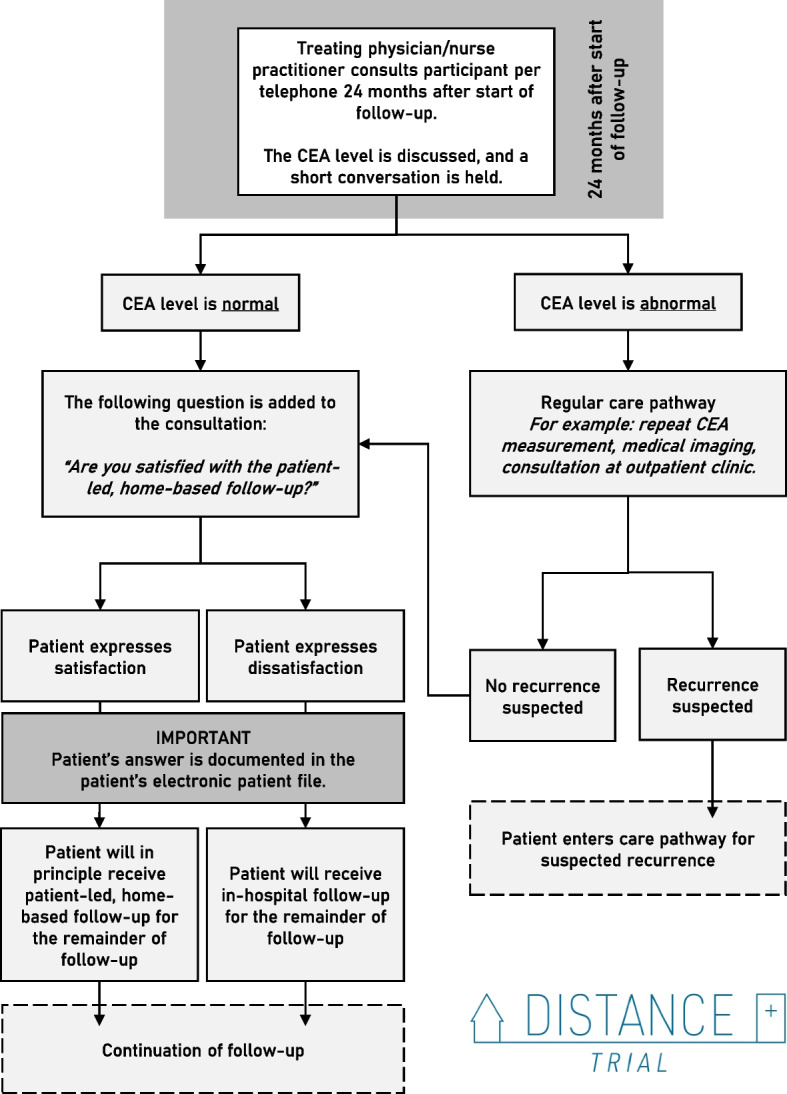
Protocol for telephone consultation at end of study. CEA; carcinoembryonic antigen

At 24 months after start of the follow-up, a telephone consultation is planned. During this regular telephone consultation, the treating physician/nurse practitioner communicates the CEA level to the participant, and consults if the participant has any symptoms or questions. When the regular contents of the consultation have been addressed, the treating physician/nurse practitioner asks the participant the question: “Are you satisfied with the patient-led, home-based follow-up?” If a participant addresses clear objections to the PHFU, the remainder of the CRC follow-up for this participant will consist of in-hospital, standard-of-care follow-up. Otherwise, the PHFU is continued. A graphical representation of the protocol for the telephone consultation at 24 months after start of the follow-up (i.e., 12 months after inclusion in the study) has been added as Fig. 3.

### Outcomes

#### Primary outcome

The primary outcome will be the proportion of participants who had contact with the hospital regarding CRC follow-up between 12 and 24 months after curative surgical resection. This endpoint will be compared between participants included under condition A and condition B.

#### Secondary outcomes

The secondary outcomes resulting from questionnaire data will be measured at 12 months after curative surgical resection (baseline), 18 months (6 months after inclusion) and 24 months (12 months after inclusion, end of study). The following secondary outcomes result from questionnaire data:

- HRQoL, measured by the European Organisation for Research and Treatment of Cancer Quality of Life (EORTC QLQ-C30) Questionnaire;
- Fear of cancer recurrence, measured by the Cancer Worry Scale (CWS) questionnaire;
- Anxiety, depression and distress, measured by the Hospital Anxiety and Depression Scale (HADS) questionnaire;
- Utility measure, measured by the EuroQol 5-Dimension 5-Level (EQ-5D-5L) questionnaire;
- Extramural costs, measured by a selection of relevant questions from the Medical Consumption Questionnaire (iMCQ) from the institute of Medical Technology Assessment;
- Productivity losses, measured by the Work Ability Index (WAI) questionnaire.

The following secondary outcomes result from other data than questionnaire data, and will be measured over the total study period:

- Cost-effectiveness, evaluated by integration of the questionnaire data and data extracted from the electronic patient records;
- OS, evaluated by data on vital status from the NCR; and
- Cancer-specific survival, evaluated by data from the NCR on vital status and electronic patient records.

All abovementioned parameters will be compared between patients included under condition A and condition B.

#### Other variables

The following other variables will be collected from the electronic patient files and the NCR:

- Age at diagnosis;
- Date of resection;
- Date of inclusion;
- Centre of inclusion;
- Follow-up condition of centre at moment of inclusion (A or B);
- Participation in PHFU (yes or no);
- Sex (male or female);
- Localisation of primary tumour (according to the 3^th^ edition of the International Classication of Diseases for Oncology);
- Clinical and pathological TNM classification (according to the 8^th^ edition of the TNM classification);
- Type of surgical procedure (e.g., hemicolectomy, low anterior resection, etc.);
- Systemic/radiation treatment;
- Number of relevant comorbidities (according to the Charlson Comborbidity Index);
- CEA measurements; and
- Medical imaging reports.

### Participant timeline

This study starts 12 months after curative surgical resection. At this moment, CRC follow-up includes a diagnostic CT thorax-abdomen, colonoscopy, a CEA measurement with the results discussed in an outpatient clinic visit. Only patients who are disease-free at 12 months after curative surgical resection, are eligible for inclusion. Patients receive follow-up care according to condition of the hospital (A or B).

#### In-hospital, standard-of-care follow-up (condition A)

Participants who are included under condition A, will receive the current standard-of-care follow-up of the participating centre. It is possible that this current follow-up schedule deviates slightly from the current national guideline.

#### Patient-led, home-based follow-up (condition B)

Participants who are included under condition B, will receive the following follow-up care between 12 and 24 months after curative surgical resection, according to the PHFU care schedule:

- CEA measurement every three to six months (according to the national guideline);
- Outpatient clinic visit/telephone consult when requested by patient or treating physician/nurse practitioner.

The treating physician/nurse practitioner will seek contact with the patient in the following situations:

- The patient has abnormal CEA levels; or
- The patient has not performed a CEA measurement within one month of the proposed check-up date.

The treating physician/nurse practitioner will request an in-hospital outpatient clinic visit in the following situations only:

- The patient has reported symptoms;
- The patient has an abnormal CEA level according to the national guideline.

The patient can seek contact with their treating physician/nurse practitioner at any time. The patient can also request an in-hospital outpatient clinic visit with their treating physician/nurse practitioner at any time.

#### Deviation from patient-led, home-based follow-up

After the centre has switched to condition B, PHFU is the new standard-of-care in a participating centre. For significant personal reasons, it is possible to deviate from PHFU and to provide the patient with the in-hospital, standard-of-care follow-up. Clear notation of the reason for the deviation needs to be documented in the electronic patient file of this patient. Patients who deviate from the condition of the participating centre but do meet the eligibility criteria, will be analysed following the intention-to-treat principle.

If a patient does not fit in the regular in-hospital follow-up at 12 months after curative surgical resection because of medical reasons, the patient meets an exclusion criterium and can therefore not be included in the study.

#### After the study

When participants reach the end of the study period, they will in principle receive PHFU for the remainder of their CRC follow-up. Also, patients will receive a colonoscopy following the Dutch guideline on endoscopic surveillance, three years after having received their first surveillance colonoscopy. Outpatient clinic visits are only performed when requested by the patient or the treating physician/nurse practitioner.

#### Questionnaires

Participants receive different questionnaires. Depending on the preference of participants, questionnaires are sent out either digitally or by mail. Participants receive these questionnaires from PLCRC following a predefined schedule. This schedule is as follows:

- Three, six, 12, 18, 24 months after inclusion in PLCRC;
- After 24 months, every 12 months.

### Sample size

In this SW-CRT, it is expected that the primary endpoint, the proportion of patients who had contact with the hospital regarding CRC follow-up care within the 12 months follow-up period, is approximately 100% in the in-hospital, standard of care follow-up and approximately 40% during the PHFU.

Over a realistic range of assumptions, this design has at least 80% power to detect a meaningful difference in the proportions of patients contacting the hospital. Specifically, this is the case:

- If at least 20 patients per six months per hospital can be included in the DISTANCE trial;
- If the hospital intra cluster coefficient (ICC) of the primary outcome is no more than 0.20 (typical hospital ICC are around 0.05–0.10) [12]; and
- If the difference between the primary outcome in condition A and condition B is more than 40% (expected difference based on implementation in Radboud university medical center: 70%).

Of note, if 20 patients per hospital per half year will be included, then the total number of patients will be 360. Of these 360 patients, 180 will be included under condition A and 180 will be included under condition B.

### Recruitment

Around diagnosis, CRC patients are asked to be included in PLCRC by their treating physician. Patients can give their informed consent for different aspects of PLCRC (use of medical data, PROM questionnaires, additional blood samples, new studies, and information on diagnostic coincidence findings). One of the major advantages of PLCRC is that when patients have given their informed consent for PLCRC, they can participate in all studies that make use of the PLCRC infrastructure. During the outpatient clinic visit at 12 months after curative surgical resection, patients can still be recruited for and included in PLCRC.

Patients do not have a choice whether or not they are included in the in-hospital, standard of care follow-up or the PHFU; this is decided by the condition in which the participating centre is at the moment of inclusion. Once PHFU is implemented in a participating centre, this is the new standard of care for all CRC patients in follow-up in that centre. Only patients who are included in PLCRC, are eligible to be analysed in this study. Informed consent for this study specifically is not necessary.

Eligible patients are automatically included in this study. The principal investigator of the participating centre will keep track of the patients who are included in the study.

### Allocation of switch moment

Follow-up care will be provided according to the current follow-up condition of the participating centre. For three of the six participating centres, this switch to including patients under condition B will occur after six months of including patients under condition A. For the other three participating centres, the switch to condition B will occur after 12 months of including patients under condition A.

The moment of switching will be allocated using a simple randomisation based on a single sequence. The randomisation will be performed between the six participating centres before the start of inclusion by the study coordinator and the project leader.

### Blinding

Due to the study design, blinding is not possible.

### Data collection methods

Data from PLCRC, the Netherlands Comprehensive Cancer Organzsation (IKNL) and data from electronic patient files will be merged before data analysis. These data from the electronic patient files can be gathered due to the informed consent of patients for PLCRC. During the DISTANCE-trial, these data will be stored in an electronic case report form using the Castor Electronic Data Capture system. The data from the electronic patient files will be locally collected in the participating centres by the study coordinator. The likelihood of loss to follow-up is very low due to the nature of the study design, as patients will likely receive follow-up in the participating centre for the remainder of their follow-up trajectory.

### Data management

All data from PLCRC and IKNL are coded with a study number. These study numbers will be linked to the data extracted from the electronic patient files and will be stored in a pseudonymised manner. As far as possible, international ontologies and classifications will be used to code the data.

During the trial, only members of the research team have access to the data collected for this study. The metadata and raw dataset of the DISTANCE-trial will be made findable for subsequent research through the DANS EASY repository using the Radboud university medical center’s Research Information System (RIS) once the study has ended. The data will be accessible upon reasonable request. DANS EASY assigns a digital object identifier (DOI) code to the archived dataset. This DOI code will be added to the registration in RIS.

All data, software codes, research materials, published or unpublished will be managed and securely stored for a period of 15 years. The raw dataset will be stored in a comma-separated values format. This storage will be performed in accordance with guidelines from the Association of Universities in The Netherlands (VNSU) in the digital research environment of the Radboud university medical center.

### Statistical methods

#### Primary outcome

Analysis of the primary outcome will consist of mixed model analysis. In this mixed model analysis, a random effect will be used for each hospital to account for the similarity of patients within a hospital. Fixed effects will be used for follow-up condition and recruitment period.

As the primary outcome of interest is a difference in proportions on hospital level, a linear mixed model will be used (identity link).

Fit of the model on the hospital and recruitment period averages will be assessed using residual plots and observed versus predicted plots. If the fit is not considered acceptable, improvement of fit will be sought using a generalised mixed model and varying the following choices:

- Difference in proportion (identity link), odds ratio (logistic link) or ratio (log link);
- Error distribution (normal distribution, binomial distribution or Poisson);
- Adjustment for hospital level characteristics (e.g., academic, teaching, community), patient and tumour level characteristics.

#### Secondary outcomes

As the PROMs are (semi-)continuous, their analysis will consist of a linear mixed model analysis. This mixed model analysis will have a random effect for each hospital, to account for the similarity of patients within a hospital. It will also have a random effect for patients within each hospital, to account for repeated measures within a patient. Fixed effects will be included in the analysis for follow-up condition, recruitment period, patient characteristics and tumour characteristics [14]. Model fit will be assessed similarly.

The EQ-5D-5L will be used to measure quality adjusted life-years (QALYs). The scores will be transformed into health utilities using the Dutch tariff [22]. The cost-effectiveness evaluation will be carried out according to the Dutch handbook for health-economic evaluations [23]. Analysis will be conducted in agreement with the intention-to-treat principle, from a societal perspective. The time horizon will be 12 months.

Healthcare costs, out-of-pocket costs of the patient and cost in the other sectors will be included (e.g., productivity losses). Hospital resource use will be taken from the hospital records, and will include all diagnostic testing, hospital visits, telephone and email consultations. Medical resource use outside the hospital, as measured using an adapted version of the iMCQ, will provide information on visits to the GP or use of paramedical care. The WAI will provide information on productivity losses due to follow-up visits. The cost associated with resource use will be derived from the Dutch handbook for costing, and tariffs and prices published by the Dutch Healthcare Authority [24]. Total costs will be calculated by multiplying resource use by integral cost prices.

As the time horizon is 12 months, neither costs nor effects need to be discounted. Incremental costs per QALY – the incremental cost-utility ratio (ICUR) – will be calculated with their 95% confidence intervals using 5,000 bootstrap replications, which will be projected on a cost-utility plane. In addition, ICUR acceptability curves will be presented and sensitivity analysis will be performed focusing on uncertainty surrounding most important cost items. To correct for potential baseline differences in patient population between the current care and the intervention group, covariate adjustment of the cost and QALY difference will be carried out.

### Research ethics approval

This study protocol was evaluated by the Medical Research Ethics Committee (MREC) Oost-Nederland (2020–7105). It was judged that the present study was not subject to the Medical Research Involving Human Subjects Act (WMO). Thereafter, the conduct of the present study was approved by the institutional review boards of the participating centres (Bernhoven Hospital, Catharina Hospital, Gelre Hospitals, Jeroen Bosch Hospital, Rijnstate Hospital, University Medical Center Utrecht). Moreover, the conduct of this study will be according to:

- The principles of the Declaration of Helsinki, The Code of Conduct for Responsible Use of Human Tissue;
- The Code of Conduct for Responsible Use of Human Tissue (in Dutch: Gedragscode Goed gebruik van lichaamsmateriaal);
- The Medical Treatment Contracts Act (in Dutch: Wet op de Geneeskundige Behandelovereenkomst);
- The General Data Protection Regulation (in Dutch: Algemene verordening gegevensbescherming);
- The Quality Assurance for Research Involving Human Subjects (in Dutch; Kwaliteitsborging Mensgebonden Onderzoek);

### Protocol amendments

Protocol amendments will be shared with the principal investigators and the institutional review boards of the participating centres.

### Consent or assent

One of the inclusion criteria is that patients are included in PLCRC [19]. Participants of the PLCRC cohort have always given informed consent that their medical data relevant to answer research questions can be collected by members of the PLCRC research team. Moreover, participants can give additional informed consent to receive PROMs questionnaires.

### Declaration of interests

The authors declare that they have no known competing financial interests or personal relationships that could influence the trial.

### Dissemination policy

The project leaders, members of the project team and all principal investigators of the participating centres will be part of each publication. All other contributors who have contributed sufficiently (i.e., according to the CRediT guidelines) to a publication, will be part of that publication. The use of professional writers is not intended.

### Other

Because this study is not subject to the WMO, several aspects of the study protocol are not applicable to this study. This comprises the following aspects:

- Installation of a data monitoring committee;
- Conduct of interim analyses and composition of a stopping guideline;
- Collection, assessment and management of adverse events (i.e., harms);
- Auditing trial conduct; and
- Ancillary and post-trial care or compensation for those suffer from trial participation.

## Discussion

This stepped-wedge cluster-randomised trial aims to successfully implement patient-led, home-based follow-up in six hospitals in The Netherlands, and its results will provide information on the success of this implementation. The study was designed as a pragmatic trial; the study respects the protocols in place in the participating centres for the inclusions in the in-hospital, standard-of-care follow-up, but will also accept variation in the frequency of blood tests and medical imaging as permitted by the Dutch guideline [12]. The chance of successful implementation is herewith presumably increased. Furthermore, it is therefore more likely that the results from this study will reflect actual clinical practice. The results of the DISTANCE-trial will provide evidence on whether or not PHFU for stage I-III CRC treated with curative surgical resection could become the novel standard-of-care for oncological CRC follow-up.

## Trial status

Inclusion of patients started in July of 2022.

### Supplementary Information

**Additional file 1.**

## Data Availability

The metadata and raw dataset of the DISTANCE-trial will be made findable for subsequent research through the DANS EASY repository using the Radboud university medical center’s Research Information System (RIS) once the study has ended. The data will be accessible from the corresponding author upon reasonable request.

## Abbreviations

CEA: Carcinoembryonic antigen
CRC: Colorectal cancer
CT: Computed tomography
CWS: Cancer Worry Scale
DOI: Digital object identifier
EORTC: European Organisation for Research and Treatment of Cancer
HADS: Hospital Anxiety and Depression Scale
HRQoL: Health-related quality of life
ICC: Intra cluster coefficient
ICUR: Incremental cost-utility ratio
IKNL: Netherlands Comprehensive Cancer Organization
iMCQ: Medical Consumption Questionnaire
MREC: Medical Research Ethics Committee
NCR: Netherlands Cancer Registry
NTR: Dutch Trial Register
OS: Overall survival
PHFU: Patient-led, home-based follow-up
PLCRC: Prospective Dutch CRC cohort
PROM: Patient reported outcome measure
QALY: Quality-adjusted life years
RIS: Research information system
RR: Relative risk
SW-CRT: Stepped-wedge cluster-randomised trial
VNSU: Association of Universities in The Netherlands
WAI: Work Ability Index
WMO: Medical Research Involving Human Subjects Act
ZonMw: The Netherlands Organisation for Health Research and Development
